# Dietary and pharmacological modification of the insulin/IGF-1 system: exploiting the full repertoire against cancer

**DOI:** 10.1038/oncsis.2016.2

**Published:** 2016-02-15

**Authors:** R J Klement, M K Fink

**Affiliations:** 1 Department of Radiation Oncology, Leopoldina Hospital Schweinfurt, Schweinfurt, Germany; 2 Onkologische Praxis, Fürth, Germany

## Abstract

As more and more links between cancer and metabolism are discovered, new approaches to treat cancer using these mechanisms are considered. Dietary restriction of either calories or macronutrients has shown great potential in animal studies to both reduce the incidence and growth of cancer, and to act synergistically with other treatment strategies. These studies have also shown that dietary restriction simultaneously targets many of the molecular pathways that are targeted individually by anticancer drugs. The insulin/insulin-like growth factor-1 (IGF-1) system has thereby emerged as a key regulator of cancer growth pathways. Although lowering of insulin levels with diet or drugs such as metformin and diazoxide seems generally beneficial, some practitioners also utilize strategic elevations of insulin levels in combination with chemotherapeutic drugs. This indicates a broad spectrum of possibilities for modulating the insulin/IGF-1 system in cancer treatment. With a specific focus on dietary restriction, insulin administration and the insulin-lowering drug diazoxide, such modifications of the insulin/IGF-1 system are the topic of this review. Although preclinical data are promising, we point out that insulin regulation and the metabolic response to a certain diet often differ between mice and humans. Thus, the need for collecting more human data has to be emphasized.

## Introduction

It is increasingly recognized that not only age but also the denaturalization of our food, lifestyle and environment are partly responsible for the current rise in non-communicable diseases such as obesity, type II diabetes mellitus (T2D) and related types of cancer.^1, 2, 3, 4^ This has motivated research into lifestyle interventions and drugs for prevention and treatment of these diseases. One of the most promising interventions is dietary restriction (DR) of either calories in general or specific macronutrients, as it consistently has been shown to prolong life- and healthspan in a broad range of model organisms and possibly humans, too, when compared with unrestricted food intake.^5, 6^ DR targets whole-body metabolism, impacting hormones, metabolic substrates and molecular signaling pathways that have a role in metabolic disorders such as obesity and T2D. Intriguingly, the same pathways are increasingly implicated in the development and growth of cancer, as more and more associations and parallels between obesity, T2D and an abnormal metabolism of cancer patients become evident. An important example is insulin resistance: reduced glucose uptake in cancer patients compared with healthy controls during an euglycemic hyperinsulinemic glucose clamp has commonly been observed not only during^7, 8^ but also before weight loss or malnutrition.^8, 9, 10^ This has been linked to chronic low-level inflammation induced by pro-inflammatory cytokines released by the tumor and tumor-associated macrophages (see review on cachexia in the same issue of this topical issue). Along these lines, insulin resistance in T2D and obesity seems connected to low-grade chronic inflammation induced by an increased release of pro-inflammatory cytokines from predominantly visceral adipose tissue and its associated immune cells, combined with a decreased release of the insulin-sensitizing hormone adiponectin.^11^ Furthermore, hyperglycemia itself, resulting from insulin resistance, induces a pro-inflammatory environment through its effect on immune cells.^12, 13, 14^ Together, the pathological features of T2D—notably elevated serum concentrations of inflammatory cytokines, glucose, insulin and free insulin-like growth factor-1 (IGF-1)—provide a pro-tumorigenic environment that may account for the increased risk of diabetic and obese patients for the development of cancer at various sites^11^ as well as the worse prognosis of patients with cancer that display one or more of these abnormalities.^15, 16, 17, 18, 19, 20, 21, 22, 23, 24, 25^

Although inflammation is a powerful driver of tumor growth,^26^ it is the aim of this review to focus on the connection between insulin/IGF-1 signaling and cancer, and discuss possibilities to modulate these interactions through DR and pharmaceutical interventions to improve cancer outcomes. We here refer to DR as any intervention that either restricts the total amount of energy consumed without changing the macronutrient ratio (calorie restriction; CR) or restricts a particular macronutrient without necessarily lowering the energy content of the diet. Usually, CR involves a 20–50% reduction in energy intake while maintaining sufficient intake of essential vitamins and minerals.^27^ It can be achieved via chronic energy restriction, the most extreme form of which is short-term starvation (STS, corresponding to water-only fasting) or intermittend fasting (IF) regimes such as only eating every other day.

## Insulin, IGF-1 and cancer

### Molecular pathways

**Figure 1:**
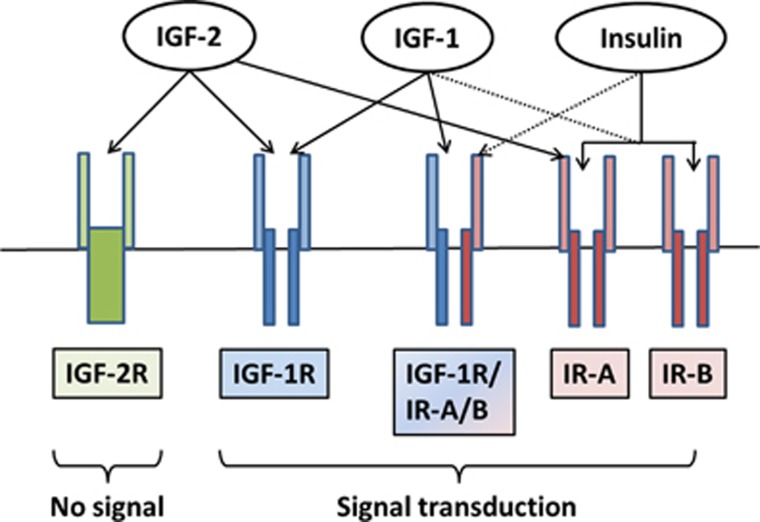
Insulin/IGF receptor binding. As tyrosine kinase receptors, the IR and the IGF receptors, consist of an extracellular ligand-binding domain and a cytosolic tyrosine kinase domain that autophosphorylates upon ligand binding and transphosphorylates several substrates that initiate downstream signaling. The IR shares ~50 and 80% homology with the ligand-binding and tyrosine kinase domain, respectively, of the IGF-1 receptor (IGF-1R).^30^ It exists in two isoforms, IR-A and IR-B, which promote either mainly mitogenic or metabolic effects, depending on the ligand and the cellular context, allowing cells flexibility in responding to mainly one or the other stimulus. In general, IR-A is preferentially associated with mitogenic and anti-apoptotic signaling, whereas IR-B is associated with cell differentiation and metabolic effects.^30^ A predominant expression of IR-A has correspondingly been found in fetal tissue and tumors with autocrine production of IGF-2, which binds this receptor with 30–40% affinity compared with insulin.^210^ In this way, these tumors promote cell proliferation in an autocrine manner.^30, 211^ IGF-2 also binds to the IGF-1R, whereas IGF-1 binds to its own IGF-1R and to hybrid receptors of IGF-1R and IR-A as well as IGF-1R and IR-B.^30, 212^ Physiological concentrations of insulin show no measurable binding to the IGF-1R both *in vitro*^30^ and *in vivo*.^213^ Nevertheless, in mammals, insulin may be the major controller of insulin/IGF-1 action due to its effect on the bioavailability of IGF-1.^43^

Insulin and the IGFs, IGF-1 and IGF-2, are structurally similar peptides with important roles in controlling metabolism and growth in response to nutrient signals and nutritional status. IGF-1-1 and IGF-2 are primarily produced in the liver and to a lesser extent locally in target tissues where they exert autocrine and paracrine actions. Both have similar biological effects that are caused by binding to the IGF-1 receptor (IGF-1R), whereas the IGF-2R is specific for IGF-2 and thought to have no physiological role except serving to degrade IGF-2^28^ (Figure 1). Serum concentrations of IGF-2 increase during childhood and then level off at ~500 ng/ml,^29^ although abnormal IGF-2 concentrations can occur in certain conditions such as IGF-2-producing tumors.^30^ IGF-1 is low at birth, increases throughout puberty and declines with older age, its concentration being roughly three times lower than that of IGF-2.^29^ Bioavailability of IGFs is regulated by a class of six IGF-binding proteins (IGFBP-1–6), which are also produced in the liver. Production of IGF-1 and the most abundant binding protein in plasma, IGFBP-3, occurs via growth hormone-mediated signaling, and insulin influences IGF-1 bioavailability by controlling the transcription of IGFBP-1.^31^

Insulin is a key hormone for coordinating nutrient intake with energy production and storage through both excitatory and inhibitory actions. Insulin is secreted from pancreatic β-cells with blood glucose being the main secretagogue in humans. As an anabolic hormone, insulin accelerates glucose uptake in various tissues and promotes lipid synthesis in the liver while simultaneously inhibiting lipolysis, proteolysis, glycogenolysis, ketogenesis and gluconeogenesis. The latter function is particularly important for insulin's well-known ability to lower and regulate blood glucose levels within a narrow physiological range during the postprandial phase.^32^

**Figure 2:**
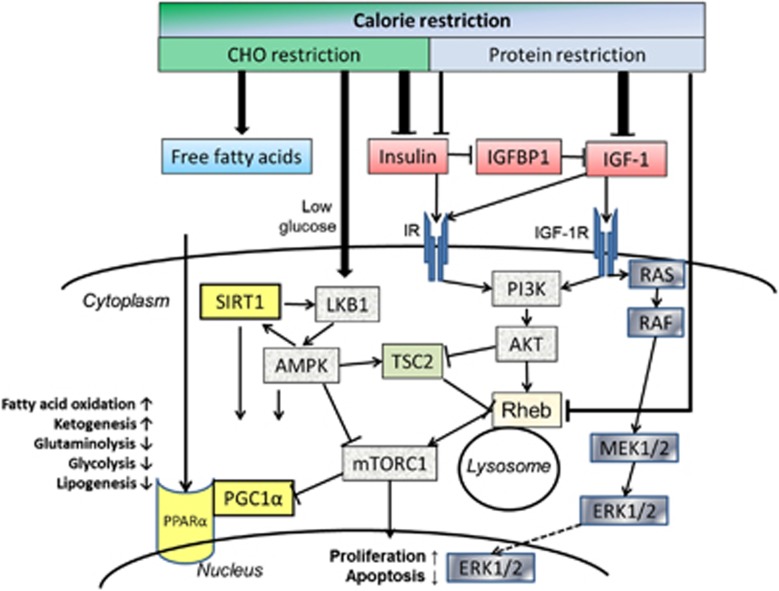
Insulin/IGF-1 signaling network and its modulation by dietary restriction. Dietary restriction in the form of overall calorie restriction or specific restriction of carbohydrates or protein has specific effects on the insulin/IGF-1 system that transduces cellular signals through its insulin and IGF-1 tyrosine kinase receptors. This picture can only provide a partial overview of the complexity of this signaling network. The classical action of activated extracellular signal-regulated kinase (ERK)-1 and ERK-2 is their translocation into the nucleus where they activate mitogenic transcription factors. Similarly, mTORC1 targets transcription factors that increase proliferation and counteract apoptosis. Activation of mTORC1 via IR/IGF-1R−PI3K−AKT converges with its activation by amino acids at the lysosomal membrane. There, the guanosine triphosphatase (GTPase) Rheb (Ras homolog enriched in brain) stimulates mTOR activity, whereas a lack of growth signals activates the tumor suppressor tuberin (TSC2), which translocates to the lysosomal membrane and inhibits Rheb-stimulated mTORC1 activation.^214^ High insulin levels activate AKT that phosphorylates and inactivates TSC2, whereas CR or glucose withdrawal induce energy stress, decrease the intracellular ATP/AMP ratio and activate TSC2 through liver kinase B1 (LKB1)—adenosine monophosphate-activated protein kinase (AMPK) signaling. AMPK can also directly inhibit mTORC1 by phosphorylating the regulatory-associated protein of mTOR (Raptor). AMPK has similar actions to the class III histone deacetylase SIRT1, which is a NAD^+^-dependent enzyme that is also activated under DR-induced energy stress through an increase in the NAD^+^/NADH ratio.^36^ AMPK and SIRT1 amplify each other and both activate the peroxisome proliferator-activated receptor gamma 1α coactivator (PGC-1α) protein that cooperates with peroxisome proliferator-activated receptor α (PPARα) to induce major metabolic shifts under DR such as an upregulation of lipid oxidation and downregulation of glycolysis.^35^ mTORC1 inhibits these actions, providing another link to insulin/IGF-1 signaling.

Insulin is also well established as a growth-stimulating hormone.^33^ In this respect, its effects parallel those of IGF-1 and IGF-2. This is due not only to the structural similarity between insulin and the IGFs, but also to the high degree of homology between the insulin receptor (IR) and the IGF receptors (Figure 1). The IR, IGF-1R and their hybrid receptors are expressed by most human tumors, whereby predominant expression of the IR-A isoform correlates with a poor differentiation grade.^30^ Among the pathways activated by IR and IGF-1R signaling are the RAS−RAF−MEK1/2−extracellular signal-regulated kinase (ERK)-1/2 pathway^34^ and the phosphatidylinositol-3-kinase (PI3K)−AKT−mammalian target of rapamycin (mTOR) pathway, two fundamental pathways for tumor cell proliferation and survival (Figure 2). The serine/threonine protein kinase mTOR is part of a protein complex called mTORC1, which has important roles in tumor cell growth and metabolism. Reduced glucose, insulin and IGF-1 levels or—more generally—DR activate an energy-sensing network consisting of AMPK, SIRT1, PPARα and PGC-1α with the potential to counteract tumor cell proliferation.^35, 36^ AMPK activation by anti-diabetic drugs such as metformin is currently considered a beneficial adjunct to standard cancer therapy.^37, 38^

### The insulin/IGF-1 system and tumor cell metabolism

**Figure 3:**
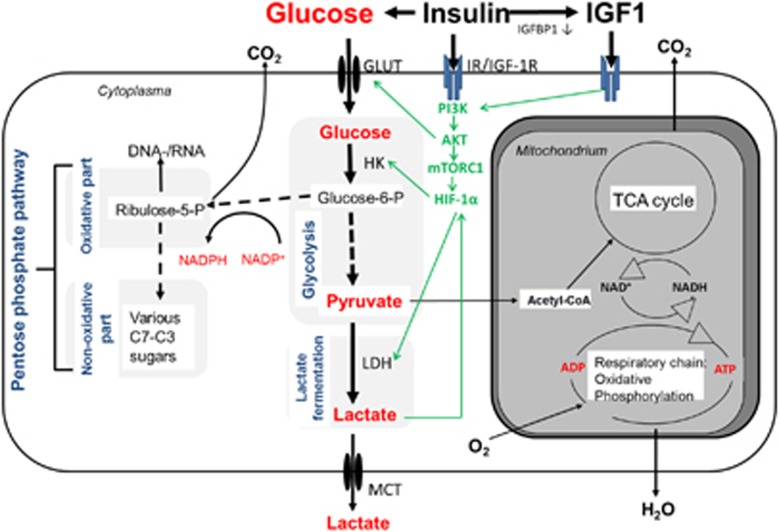
Glycolytic pathways in tumor cells. Sketch of the most important glucose-degrading metabolic pathways in a tumor cell. Glucose uptake into the cytoplasm is accomplished via specific transpcorters (GLUTs) that are often overexpressed in tumor cells. Here the enzyme hexokinase (HK) phosphorylates glucose to glucose-6-phosphate (glucose-6-P). This metabolite either gets degraded to pyruvate via several intermediate steps of glycolysis or serves as the precursor for conversion into ribulose-5-phosphate in the oxidative part of the pentose phosphate pathway (PPP). In the PPP, CO_2_ gets released and the reducing equivalent NADPH is produced. The generated ribulose-5-phosphate either serves as the basis for *de novo* synthesis of nucleotides or is converted to various C3–C7 sugars through the transketolase/transaldolase reaction in the non-oxidative part of the PPP. Pyruvate, the end product of glycolysis, usually gets transported into the mitochondria, converted to acetyl-CoA and channeled into the TCA cycle for oxidative degradation. In case of insufficient oxygenation, dysfunctional mitochondria or metabolic reprogramming through hyperactivation of AKT–mTOR signaling, pyruvate is increasingly converted to lactate via the enzyme lactate dehydrogenase (LDH). Lactate gets transported out of the cell by monocarboxylate transporters (MCTs). The PI3K–AKT pathways increases glycolytic rate by the mechanisms depicted and described in the main text. Dashed arrows indicate several intermediate steps.

Apart from a few exceptions, glucose has a key role in tumor cell metabolism and its connection to proliferation and cell protection. As known since the seminal studies of Otto Warburg *et al.*^39, 40, 41^ most tumors ferment glucose to lactate even under sufficient oxygen supply, which in normal cells of the same tissue would shuffle pyruvate, the end product of glycolysis, into the mitochondria for further oxidative utilization. This peculiar feature of tumor cell metabolism is now known as the ‘Warburg effect' or ‘aerobic glycolysis'. It is the basis of molecular 2-(18 F)fluoro-2-deoxy-d-glucose (FDG)-positron emission tomography (PET) imaging with the radioactively labeled glucose analog FDG (Figure 3). A high uptake of glucose in tumor cells not only serves for energy production but also for protection from endogenous and exogenous reactive oxygen species because NADPH, a by-product of the oxidative pentose phosphate pathway, is used to regenerate glutathione, an important cellular antioxidant.

On a molecular basis, the PI3K–AKT–mTORC1 pathway has been found to significantly contribute to the high glycolytic activity of many tumor cells.^42^ AKT directly and indirectly—by the activation of mTOR, which promotes the stabilization of the transcription factor hypoxia-inducible factor-1α—stimulates the expression of glucose transporters and key glycolytic enzymes (Figure 3). AKT also phosphorylates the pro-apoptotic and anti-proliferative transcription factor FOXO1, which leads to its exclusion from the nucleus and cytosolic degradation, thereby connecting tumor cell metabolism with cell cycle progression and survival.

High IGF-1 and insulin levels in the microenvironment therefore provide a plausible mechanism of carcinogenesis and early tumor growth through anti-apoptotic signaling and metabolic reprogramming mediated by the PI3K–AKT–mTORC1 pathway. This is consistent with the finding that diabetes and obesity mainly raise the risk for those cancers that exhibit a Warburg phenotype.^43^ The relevance of this pathway for tumorigenesis is demonstrated by the fact that humans with the Laron syndrome, a recessively inherited defect in the growth hormone receptor, display extremely low IGF-1 and reduced insulin concentrations and usually do not develop cancer despite high prevalence of obesity and dyslipidemia.^44, 45^

Human tumors stimulated by insulin *in vitro* include breast cancer,^46, 47^ colon cancer,^48^ various leukemia cells lines^49, 50, 51^ or melanomas.^52^ In addition, most cancer cells are extremely vulnerable to glucose withdrawal,^53, 54, 55, 56, 57, 58, 59^ a feature they owe to metabolic reprogramming, leading to constitutively active proliferation pathways and ‘glucose addiction'. In fact, hyperglycemia itself stimulates tumor growth through distinct mechanisms and often amplifies the growth-promoting action of insulin.^60^ Thus, 100 ng/ml of insulin increased proliferation rates of human breast, colon, prostate and bladder cancer cell lines in a glucose-dependent manner, achieving 7–44% higher proliferation when combined with diabetogenic glucose concentrations of 11 mm compared with glucose concentrations of 5.5 mm without added insulin.^61^ Furthermore, high glucose and insulin altered the activity of several cell adhesion and migration genes, increasing migratory ability and the duration of locomotion. This coincided with an upregulation of the PI3K pathway,^61^ and a 29 and 66% increase of Akt expression in MDA-MB-468 breast and SW480 colon cancer cell lines, respectively.^62^

Despite these indications of tumor sensitivity to insulin, it is not clear to which extent the modulation of insulin and IGF-1 levels is able to influence the proliferation of progressive cancers that have become self-sufficient in growth and metabolic signals as illustrated for example by: (i) expression of insulin-independent glucose transporter isoforms; (ii) concurrent overexpression of IR-A and IGF-2; (iii) gain of function mutations in the *PIK3CA* gene, encoding the catalytic subunit of human PI3K; and (iv) loss of function mutations in *PTEN*, encoding the phosphatase PTEN which inhibits PI3K. This may not only pose a resistance mechanism against specific IR and IGF-1R inhibitors but also against dietary and pharmacological insulin and IGF-1 modulation. Kalaany and Sabatini had shown in NOD-SCID (non-obese diabetic, severe combined immunodeficient) mice that xenografted tumors with constitutive activation of AKT by either gains in *PIK3CA* or loss of *PTEN* are resistant against CR, and insensitive to insulin and IGF-1 treatment *in vitro*. At the same time, tumors without such a constitutive activation were stimulated by insulin and IGF-1, and responded to CR with increased rates of apoptosis mediated through FOXO1.^63^ This is reminiscent of the classical studies of the 7,12-dimethylbenz(a)anthracene (DMBA)-induced mammary carcinoma of the rat by Heuson and Legros. In rats bearing this tumor, induction of type-1-like diabetes with alloxan was followed in up to 90% of cases by rapid onset of remission, whereas treatment with insulin and especially combined treatment with insulin and glucose stimulated tumor growth considerably.^64^ These insulin-sensitive tumors also regressed in response to 60% CR, and insulin treatment *in vitro* induced a parallel rise of DNA synthesis and DNA polymerase activity.^65^ Some tumors however had apparently reached an autonomous growth without responding to insulin withdrawal or CR, similar to the tumors with constitutively activated AKT studied by Kalaany and Sabatini.

**Figure 4:**
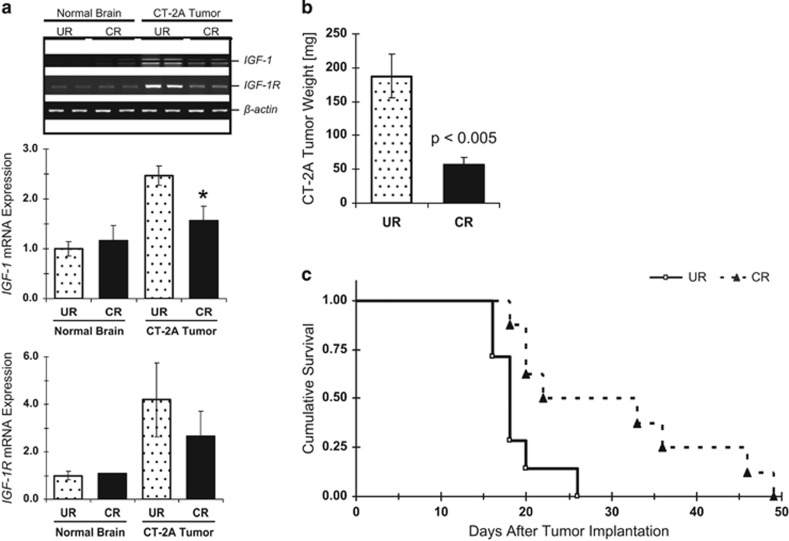
Effects of CR on IGF-1 and IGF-1R mRNA expression and growth of the CT-2A astrocytoma. For conditions **a** and **b**, tumors were implanted into the brains of C57BL/6J mice. At 10 days post tumor implantation, mice were randomly switched to either an unrestricted (UR; *n*=9) or CR (*n*=9) diet that aimed at reducing body weight by ~30%. In condition **c**, tumors were implanted subcutaneously and CR started at day 14; the Kaplan–Meier survival curve indicates significantly longer survival for CR compared with UR (*P*=0.01). Figure parts reproduced with permission from^67^.

Some inconsistencies, however, remain. For example, the U87-MG glioma that was found resistant to CR when grown as a subcutaneous xenograft in NOD-SCID mice^63^ was responsive to CR when grown orthotopically in mice of a different genetic background.^66^ The CT-2A malignant mouse astrocytoma responded to CR when grown orthotopically or subcutaneously in C57BL/6J mice despite its PTEN and TSC2 deficiency, and constitutive AKT activation.^67^ Notably, growth retardation was accompanied by decreased phosphorylation of AKT and IGF-1R/IR tyrosine kinase domains, decreased production of IGF-1 and downregulation of IGF-1R protein expression in the tumors (Figure 4).

It was further shown that in some cases, including a large percentage of human non-small cell lung cancers, AKT activity can be low despite loss of *PTEN*; in an experimental setting, such cancers were sensitive to upstream activation by insulin and IGF-1, and regressed during CR.^68^ Other common mutations in oncogenes such as *RAS* and *BRAF* or tumor suppressors such as *TP53* did not affect the sensitivity of such tumor cells to insulin and IGF-1 *in vitro* or to CR *in vivo*.^63^ Together, these findings indicate that (i) the sensitivity of tumors to insulin and IGF-1 parallels their response to CR, and (ii) neither the activation status of PI3K−AKT *per se* nor mutation status of individual genes predicts for the sensitivity of tumors to both growth factors and CR; instead, the metabolic environment of the host (NOD-SCID mice display signs of both type 1 and type 2 diabetes^69^) and genetic conformity between host and tumor (xenograft/allograft/isograft) seem to have the dominant role.

There are hints that constitutive activation of PI3K–AKT signaling could be exploited therapeutically because it underlies a differential stress response between malign and benign cells, such that only the latter increase their resistance against cytotoxic insults upon reduction of growth factors induced by CR.^70^ In C57BL/6J mice, CR promoted stress resistance in a FOXO1-dependent manner,^71^ which would not occur in tumor cells with FOXO inactivation due to constitutively active AKT. We have recently argued that a similar, albeit less pronounced, differential stress response may also be induced by a ketogenic diet (KD).^72^ A KD is usually defined as an isocaloric diet low enough in CHO and high enough in fat to induce significant elevations (⩾0.5 mmol/l in humans) of the ketone bodies (KBs) β-hydroxybutyrate and acetoacetate (termed ‘ketosis'). It therefore can be considered a fasting mimicking diet. This would be of special value for patients undergoing several week long radiotherapy during which prolonged fasting is no option. The state of ketosis, induced by low insulin levels, may generally benefit cancer patients, as KBs have been shown to inhibit glycolysis in various tumor cell lines^73, 74^ and probably also patients,^75, 76, 77^ and tumor cells often lack the enzymes^78, 79, 80, 81^ or oxygenation^82^ to effectively use ketones for energy production. Furthermore, in glioblastoma xenografts it was shown that KBs can partly reverse the genetic alterations that occur in these tumors.^83^

In conclusion, beneficial effects may be achieved by lowering insulin and IGF-1 levels in patients with both insulin-sensitive and insulin-insensitive tumors.

As a final note, some experimental tumors have been described whose growth is suppressed by insulin and stimulated by induction of type 1 diabetes, with the R3230AC adenocarcinoma of the rat being the most intensively studied tumor of this type.^84, 85, 86^ A tumor-suppressing effect of insulin and glucagon, and especially of their combination has been found by Salter *et al.*^87^ and was reproduced in different experimental systems.^88, 89^ The Morris hepatoma, which was also suppressed by insulin,^90^ was stimulated by acute fasting,^91^ and it was later discovered that linoleic and arachidonic acid released from adipose tissue were the main substrates promoting tumor growth.^92^ Similarly, in a hamster model of pancreatic cancer, high substrate levels of glucose, linoleic acid and other fatty acids mobilized through streptozocin-induced diabetes significantly enhanced tumor growth, which was prevented by insulin treatment.^93^ Collectively, these experimental tumors provide evidence not for a direct tumor growth-suppressing effect of insulin but indirect effects such as an influence on metabolic growth-promoting substrates whose global abundance is controlled by insulin.

## DR and its effect on the insulin/IGF-1 system

The importance of the insulin/IGF-1 system for the antitumor effects of DR is exemplified by the fact that *in vivo* IGF-1 administration^94, 95, 96^ completely rescued CR-sensitive tumors from CR-induced growth inhibition. Therefore, it is important to review the effects of different DR regimes on circulating levels of these growth factors and other substrates influenced by them. Relevant to many preclinical studies are the extensive investigations in male C57BL/6 mice that are summarized in Table 1 and compared with the data from insulin-resistant humans, because many cancer patients are also insulin resistant. From Table 1, it is already evident that mice and humans can respond differently to the same DR regime, an important fact we will elaborate on later.

### Calorie restriction

Because of the simultaneous lowering of all three macronutrients and energy, CR induces a complex metabolic response that is not straightforward to attribute to one of these individual factors. Mitchell *et al.*^97^ have shown that 10–40% CR over 3 months in C57BL/6 mice (corresponding to approximately a tenth of their lifespan) decreases blood glucose, insulin and IGF-1 concentrations, the latter showing the greatest dependence upon the severity of CR.

Many of the metabolic effects of CR in model organisms are also observed in humans.^5^ CR reduces fasting insulin levels and improves insulin sensitivity in overweight individuals, whereby these effects may be more pronounced with regular IF compared with chronic CR.^98, 99^ A CR diet supplying 600 kcal/day over 8 weeks significantly decreased fasting insulin from 151 to 65 pmol/l and glucose levels from 9.2 to 5.7 mmol/l in overweight individuals with T2D, with the greatest declines already apparent after 1 week.^100^ Kitada *et al.*^101^ showed that 7 weeks of 25% CR significantly reduced insulin levels and inflammatory markers in healthy obese males. Interestingly, incubation of human skeletal muscle cells with serum obtained from these subjects after the intervention resulted in an increase in PGC-1α expression, AMPK and SIRT1 activity, and mitochondrial biogenesis. Mercken *et al.*^102^ investigated tissue samples from m. vastus lateralis of individuals following long-term CR (average 9.6 years) of ~30% compared with a typical Western diet. Thereby, PI3K and AKT transcription was downregulated 1.7- and 2-fold, respectively, whereas PGC-1α transcripts were increased 7-fold and AKT phosphorylation was reduced by 35–50%. This indicates that chronic CR reduces PI3K−AKT signaling in humans.

In contrast to rodents, IGF-1 levels in humans are usually not reduced with chronic CR unless protein is also restricted^103^ (see below). STS, on the other hand, decreases IGF-1, glucose and insulin levels, and increases KB levels comparable to 40% CR over 3 weeks in mice.^104^

Clinically, it is well established that weight loss and physical activity can reduce insulin levels by 10–30%, and that lowering of insulin by 25% may be associated with a 5% absolute improvement in breast cancer mortality.^105^ Although this is not generalizable to other cancer patients, especially those at risk for malnourishment, IF and STS could be considered as therapeutic options in such cases; recommendations for their implementation are given by Simone *et al.*^106^ or Klement and Champ.^72^

### Carbohydrate restriction

The metabolic response of different mice strains to CHO restriction differs and is also influenced by the total energy intake and the percentage of the remaining macronutrients. A classical biomarker of CHO restriction is the amount of circulating KBs.

Insulin and glucagon are key hormones regulating ketogenesis by controlling the flux of non-esterified fatty acids to the liver for KB production.^107^ Paradoxically, C57BL/6 mice gain excessive body weight, display hyperlipidemia, and have concurrently elevated KB and glucose levels when fed an unrestricted KD with >10% energy (E%) from protein.^108, 109^ Thereby, insulin levels are only slightly decreased, but drop significantly together with blood glucose levels and body weight once calorie intake is also restricted.^109^ This is in contrast to healthy humans where unrestricted KDs with adequate protein intake tend to lower body weight and insulin levels,^110^ improve body composition by increasing fat-free mass^110, 111^ and decrease inflammatory markers.^112^ Some authors argue that CHO restriction should be the first approach in the treatment of T2D, as it improves long-term glucose control, lowers insulin levels and allows a reduction or even elimination of anti-diabetic drugs.^113^ Indeed, investigation of glucose kinetics and insulin secretion after a low-CHO meal (20E% CHO, 65E% fat) in five young adult baboons revealed minimal perturbations of glucose homeostasis, in stark contrast to a high-CHO (65E% CHO, 20E% fat) meal.^114^ Consistently, it has been shown that the metabolic effects of fasting in humans are largely mediated by the absence of CHO.^115^ This is the rationale for using KDs as fasting mimicking diets in the oncological setting where maintenance of fat-free mass is important.^116, 117^

### Protein restriction

Mitchell *et al.*^97^ found that isocaloric protein restriction down to 12E% over 3 months was not able to reproduce the beneficial metabolic changes induced by CR in male C57BL/6 mice, in particular the decrease in IGF-1 concentrations. Other studies, however, have reported reduced IGF-1 levels in mice of this strain when protein was restricted to <10E% within the context of either *ad libitum* high-CHO^118^ or high-fat^119^ diets. In mice fed *ad libitum* an 8-week protein restriction to 5E%^120^ or methionine restriction^121^ have been shown to mimic several of the metabolic effects of CR, such as decreases in triglycerides, blood glucose and insulin levels. In the long run, however, such diets may impair gains in lean body mass, and five out of nine experimental diets with protein restricted to 5E% turned out to be not sustainable due to excessive weight loss (>20%), rectal prolapse or failure to thrive (Supplementary Table S1 in^118^).

A change in IGF-1 concentrations is a marker for acute changes in nitrogen balance that depends on protein intake, but also on total energy intake.^122, 123, 124, 125^ Insulin, for example, inhibits protein breakdown, facilitating the maintenance of positive nitrogen balance when protein is replaced with CHO. Accordingly, IGF-1 levels in healthy humans dropped significantly during STS^122, 123^ or the initiation phase of a KD^126^ but were unaltered after several weeks of a KD^110^ or long-term CR with adequate protein intake.^127^

Another biomarker of protein restriction is fibroblast growth factor 21 (FGF21). FGF21 has originally been described as a fasting hormone that is upregulated in the liver via PPARα and partly regulates ketogenesis during starvation or KDs;^128^ an important role for SIRT1 in FGF21 expression was also recently demonstrated.^129^ However, a study using *Fgf21*-knockout mice found that FGF21 was not required for ketogenesis or other adaptions to a KD.^119^ It was later shown that it is the *de facto* protein restriction during starvation or protein-deficient diets that increases FGF21 concentrations as only diets with <10E% from protein,^128, 130^ but not those with higher protein intake,^119, 131^ led to an upregulation of hepatic FGF21 expression and secretion, independent of energy or CHO intake.^131^ This is consistent with the data in humans showing that a low-protein diet (5E% protein) increased plasma FGF21 concentrations by 171% over 4 weeks despite caloric overfeeding.^131^ Other data indicate that depletion of single amino acids such as methionine is sufficient for hepatic FGF21 production.^121^ There are data showing that FGF21 acts as an insulin-sensitizing and glucose-normalizing hormone in diabetic states and contributes to the action of anti-diabetic drugs.^132^

Protein restriction also limits mTORC1 activation by mechanisms distinct from its regulation via IR/IGF-1R−PI3K−Akt. It is generally believed that activation of mTORC1 by specific amino acids, notably leucine, starts with its recruitment to the lysosomal membrane by GTPases called Rags, which are concentrated there as heterodimers consisting of RagA or RagB combined with RagC or RagD. These heterodimers are part of an amino-acid-responsive supercomplex also containing the Ragulator and vacuolar adenosine triphosphatase protein complexes. This supercomplex in turn is thought to be activated by amino acids transported from the lysosomal lumen by transmembrane proteins, which in this way act as amino-acid sensors.^133^ Two alternative, Rag-independent ways of mTORC1 activation were also recently described. Glutamine, but not leucine, was found to activate mTORC1 by a pathway requiring the ADP ribosylation factor-1 GTPase and vacuolar adenosine triphosphatase for mTORC1 translocation and fixation, respectively, to the lysosomal membrane.^134^ Thomas *et al.*^135^ described a pathway involving amino-acid- but not insulin-stimulated binding of the small GTPase Rab1A and mTORC1 with subsequent recruitment to Golgi membranes where mTORC1 gets activated by Rheb. This study identified Rab1A as an oncogene in certain human cancers whose overexpression promotes amino-acid-stimulated tumor growth but also renders these cells vulnerable to amino-acid restriction.^135^

In humans, AMPK activation from training in a glycogen-depleted state was not influenced by protein intake.^136^ Other data have shown that CHO restriction is sufficient to activate the AMPK–SIRT1–PGC-1α network in humans even under caloric overconsumption.^137^ These findings complement the previously mentioned hormonal and metabolic changes induced by CHO restriction^115^ and indicate that in humans CHOs have a more dominant role than protein in the response to fasting.

In summary, protein restriction exerts specific effects on IGF-1, FGF21 and mTOR activity that probably contribute to the life-prolonging and anticancer effects seen when rodents are placed on low-protein diets. These effects can partly be mimicked by the restriction of certain amino acids. On the other hand, severe restriction of total protein intake, that is, either very-low-protein diets (<10E%) or moderate protein intake combined with CR, with the aim of reducing IGF-1 levels bears the risk of weight and fat-free mass loss. This would have detrimental effects for cancer patients, thus precluding severely protein restricted diets from a role as supportive interventions in cancer patients.

### DR and tumor growth retardation

#### Animal data

Two meta-analysis have evaluated the evidence for tumor growth inhibition by CR. Focusing on studies on spontaneous breast tumors in mice published between 1942 and 1995, Dirx *et al.*^138^ found that CR led to an average of 55% less tumor development in CR-fed mice than in *ad libitum* controls. In a recent meta-analysis, 40 out of 44 studies (90.9%) showed a tumor inhibitory effect of CR in laboratory animals with respect to tumor incidence, progression or metastasis.^27^ The evidence for a protective role of various IF protocols was weaker, but still mostly positive. Furthermore, eight out of nine preclinical studies evaluated in this meta-analysis showed that a KD was able to slow down tumor growth, often even as a monotherapy.^27^

In a study not included in these meta-analyses, Frimberger *et al.*^139^ not only achieved a retardation of tumor growth but in 36% of cases a complete remission of benzo(α)pyren-induced cutaneous squamous cell carcinomas. The mice in this study were placed on a maximally tolerable restriction of both calories and protein which was accompanied by an extreme loss of up to 50% body weight.

In C57BL/6 mice, an unrestricted KD with 13E% from protein reduced tumor growth after transplantation of Lewis lung carcinoma cells compared with a high-CHO diet (77E% CHO), and this became significant when protein was further restricted to 5E% and replaced with fat.^119^ In another study, a lower number of lung metastases was observed when mice of this strain bearing the B16 melanoma were fed a zero-CHO, zero-protein (100E% from polyunsaturated fatty acids) diet.^140^ In contrast, growth of the CT-2A Astrocytoma in these mice was not influenced by an unrestricted KD containing 17E% protein,^141^ but significantly reduced on a calorically restricted KD containing only 8E% protein, along with significant reductions in blood glucose and IGF-1.^142^ These findings might be correlated to the metabolic abnormalities that these mice develop on KDs that are not concurrently low in protein as discussed above.

In the majority of studies, antitumor effects of CHO restriction have been achieved without concurrent CR. In the studies that proofed tumor growth inhibition by a KD fed *ad libitum,* there was a significant increase in KB levels, but not necessarily decreases in blood glucose levels or body weight.^74, 140, 143, 144, 145, 146, 147, 148^ In some models, also non-ketogenic low-CHO diets (10–15E% CHO) led to significant tumor growth retardation that was correlated to low blood glucose and insulin levels.^149, 150^

#### Clinical data

The large preclinical support for CR as an antitumor therapy implies a possible role for CR in human cancer prevention, treatment and survivorship.^151^ However, clinical trials to test its effects in patients have only recently started, and published results of CR interventions are restricted to small pilot studies or case reports,^75, 76, 77, 152, 153, 154, 155, 156, 157, 158, 159^ which we summarize in Table 2. One study reported the successful treatment of a patient with end-stage ovarian cancer by a diet allowing only 300–400 kcal/day.^158^ Notably, bioimpedance analysis indicated that of 21 kg body weight loss within 6 months <2 kg consisted of muscle mass.

In an evaluation of 10 patients, it was found that STS before and/or after chemotherapy reduced therapy-related weakness, fatigue and gastrointestinal side effects.^152^ Importantly, fasting did not interfere with the cytotoxic effect of chemotherapy on tumor cells. This might indicate that the fasting-induced differential stress response between tumor and normal tissue may also be achieved in humans.

Seven case reports exist for the treatment of glioblastoma multiforme with a CR-KD. Although this diet achieved one remission of 5 years when used as a monotherapy,^75^ the usual progress within 12 weeks occurred in two other cases.^159^ The results obtained by combining the CR-KD with chemo- or radiotherapy had been more promising, with remissions between 4 months and 4 years having been achieved.^75, 153, 160, 161^

Ninteen more patients with recurrent glioblastoma were treated in the ERGO trial by Rieger *et al.*^157^ with a KD consumed *ad libitum.* Here, too, no clinical effects on tumor growth were achieved with the KD as a monotherapy, with a median progression-free survival of 5 weeks (range 3–13 weeks). However, subsequent salvage therapy with bevacizumab and continuation of the KD led to a response in six out of seven patients, and the authors confirmed in a mouse experiment that the combination of KD and bevacizumab, but not the KD alone, was superior to bevacizumab combined with standard chow with respect to survival and tumor size.^157^ A partial explanation for the failure of the KD as a monotherapy in the ERGO trial could be based on the fact that there was no significant drop in blood glucose or HbA1c levels, and only part of the patients reached stable ketosis.^157^ According to Seyfried *et al.*^162^ the ratio between blood glucose and KB levels (both measured in mmol/l) should be below ~1.5 to enable metabolic management of malignant brain cancer. As insulin inhibits ketogenesis, low insulin levels are a prerequisite to achieve such a metabolic state.

The importance of low insulin and high ketone body levels is also implicated by the pilot trial of Fine *et al.*^76^ in which five out of nine patients with previous progression responded to a 4-week KD with partial remission or stable disease as judged by FDG-PET scans; these five patients exhibited significantly higher KB levels compared with their baseline values than the four non-responders. Ketosis was thereby inversely correlated to insulin levels. Together with the other reports measuring less FDG uptake or a decline in intratumoral lactate levels as summarized in Table 2, this implies that a KD is able to influence tumor cell metabolism by lowering insulin levels and increasing ketone body concentrations.

In addition, small parenteral feeding studies obtained direct hints that a lipid-based diet (fat contributing 80% non-protein calories) retards tumor cell proliferation while a dextrose-based diet (dextrose contributing 100% non-protein calories) accelerates it.^163^ A mixed diet (fat contributing 45% non-protein calories) investigated in another study had no effect on tumor cell kinetics.^164^ Finally, some, but not all, studies found worse survival in patients who received nutritional support in the form of high glycemic supplements^165^ or total parenteral nutrition.^166^ This could be taken as a warning to monitor the caloric and glycemic load of the diet to avoid overnutrition of patients that would resemble the feeding of control animals in the preclinical DR studies.

### Critically questioning the concept of DR: implications for humans

Together, it seems that humans are more sensible to the amount and quality of CHO in their diet than mice in which high-protein intake can decisively stimulate insulin output.^108, 118^ This is not surprising, given that the species-specific diet in mice is very different from humans who are omnivores and during much of their evolution consumed low-glycemic load diets.^167^ Furthermore, mice and humans differ substantially in insulin kinetics and blood glucose control.^69, 168^ Expression of mouse carbohydrates and lipids on xenografted human tumor cells has also been described and could potentially alter the response of such tumors to the metabolic microenvironment.^169^ Together, these findings question the relevance of such tumor models for humans and complicate the translation of interventions tested in mice to human subjects.^170^

This also applies to DR: a thorough investigation of the life-prolonging effects of DR in mice shows that weight gain in the *ad libitum* fed control group is the most important covariate explaining most of the variation in the response of different strains to DR.^171^ Making the reasonable assumption that the mechanisms behind the life-prolonging effects of DR are also responsible for its antitumor action, one would have to conclude that DR in humans is most effective if it could replace a diet leading to weight gain and metabolic disturbances when consumed *ad libitum*.^117^ The prototype of such a diet is the Western diet, which is concurrently high in refined CHOs and fat. Thus, it could be expected that CR, CHO restriction or other fasting mimicking diets^172^ could be effective against tumor growth in humans. It must be considered, however, that mice have a metabolic rate approximately seven times higher than humans, and their experimental tumors display faster doubling times and larger relative weights.^173, 174^ Contrary to mice that appear fit and viable even with very high tumor masses, humans generally develop advanced cachexia and die when tumor masses have already reached 0.1% of body weight.^174^ Furthermore, relative weight loss in rodents under a particular DR regime is more rapid and extreme than in humans, with up to 50% weight loss being tolerated in tumor-bearing animals.^139^ Thus, the effects of any DR regime are exacerbated in mice, explaining why DR as a monotherapy has worked in preclinical studies but not in humans. Studying humans is therefore urgently needed to determine the dose–effect relation of DR interventions in human cancer treatment.

## Pharmaceutical interventions

The modulation of plasma insulin and IGF-1 levels by pharmaceutical interventions is a promising approach for cancer treatment. Broad interest is now focused on the biguanide metformin^37, 38^ (see review in the same issue of this topical issue) as well as IR and IGF-1R inhibitors.^37^ The α-glucosidase inhibitor acarbose, which suppresses several tumors, but simultaneously promotes kidney tumors of the Sprague Dawley rat,^175^ has been recently reviewed.^176^ As an extensive discussion of these drugs is beyond the scope of this review, we refer the reader to the referenced work and focus our discussion on two less frequently mentioned interventions for which preclinical and clinical experience has been made by one of us (MKF). These are the administration of insulin itself to potentiate the effect of chemotherapy, and administration of the insulin-lowering drug diazoxide.

### Insulin administration for cancer treatment

Although somewhat counterintuitive, reports have been published suggesting that insulin can be used therapeutically to treat cancer under certain conditions. One approach is to utilize the glucose-lowering effects of insulin to withdraw this preferred metabolic substrate from cancer cells. The idea is not new, as already Otto Warburg *et al.*^177^‘…kept tumor animals in very low blood sugar content in insulin convulsions for hours'. Although these experiments had no effects on tumor viability, the concept was later picked up by Wilhelm Brünings^178^,^179^ who combined maximally tolerable doses of insulin treatment with a KD into a ‘de-glycation method' (‘Entzuckerungsmethode') for the treatment of head and neck cancer patients in his clinic. The results, published in 1941/1942, indicated a very high rate of partial and complete remissions after a few weeks of treatment, but tumors became refractory after 2–3 months. Although the results could not be replicated by others,^180^ 15 years later Joseph Weiss^181^ was able to achieve significant tumor growth inhibition in 20 out of 90 incurable cancer patients with a similar method. Finally, two case reports exist according to which repeatedly administered high doses of insulin resulting in hypoglycemic coma (lowest blood glucose reading 22 mg/dl) were able to bring metastatic cancer in complete remission of at least 1-year duration.^182^

A second approach using insulin administration consists of giving low dosed chemotherapeutic drugs at onset of hypoglycemia after intravenous administration of (typically 0.3–0.4 IU/kg) insulin, followed by hypertonic glucose. This so-called insulin potentiation therapy is hypothesized to increase drug uptake into tumor cells and additionally sensitize them to the chemotherapeutic substances through insulin's ability to accelerate cell cycle progression into S-phase.^183^ However, *in vitro* both of these mechanisms were not responsible for insulin's drastic enhancement of cytotoxicity of the folic acid analog methotrexate to MCF-7 human breast cancer cells.^184^ But, in a preclinical study on the DMBA-induced rat mammary carcinoma, this enhanced effectiveness of methotrexate was far outweighted by the growth-stimulating effect of 3-day pretreatment with combined insulin/glucose infusions.^185^ In a clinical pilot trial on 14 advanced cancer patients, combined insulin/glucose infusions were therefore started at most 18 h before the administration of methotrexate/5FU.^186^ Although results were mixed regarding an increased efficacy of combined treatment, there were some indications that tumor-associated pain could be reduced, possibly due to insulin's anti-inflammatory action.^186^ Currently, insulin potentiation therapy is practiced by over 400 therapists worldwide, and the few data available indicate that it could allow a reduction of chemotherapeutic doses without compromising efficacy.^187^

### Reduction of insulin levels with diazoxide

Diazoxide is a nondiuretic benzothiadiazine that has an antihypertensive effect and produces hyperglycemia via lowering insulin levels by activation of ATP-sensitive K^+^-channels, which have a key function in the control of insulin release.^188, 189^ In addition, diazoxide stimulates insulin degradation in the lysosomal system.^190^ With 300 mg diazoxide per day, fasting insulin levels decreased from 177 to 123 pmol/l (*P*<0.01), and insulin release in response to 100 g oral glucose administration decreased from 223 to 55.6 nmol × min/l (*P*<0.002) in obese patients with polycystic ovary syndrome.^191^ This effect was less pronounced in healthy non-obese women with a decrease of insulin release from 108 to 49.3 nmol × min/l (*P*=0.05).^192^ In moderately overweight patients with polycystic ovary syndrome, 300 mg diazoxide per day also reduced IGF-1 from 314.5 to 219.5 ng/ml (*P*<0.01).^193^ Furthermore, diazoxide inhibits glucagon secretion in healthy man^194^ and in the dog,^195^ but stimulates glucagon release in rats.^196^

Typical oral doses of diazoxide for the treatment of patients suffering from hyperinsulinemia^197, 198^ and resistant hypertension^199^ were 400 mg per day, with maximal doses of 800^199^ and 1500 mg/day,^197^ respectively. The main recognized side effects of orally given diazoxide are fluid retention, nausa and the growth of lanugo hair.^197, 198, 199^

The effect of diazoxide on cancer growth was examined in DMBA- and N-methyl-N-nitrosurea (MNU)-induced mammary carcinomas of the rat.^200, 201^ The determination of glucose and insulin levels in the blood of DMBA-induced tumor-bearing animals showed that tumor induction itself led to significantly higher glucose and lower insulin levels than in control animals. Increasing dosages of diazoxide led to an increasing number of remissions. After cessation of diazoxide treatment due to progression, 30% rebound responses were observed in animals that had a first remission due to diazoxide. This second remission after withdrawal of the drug is characteristic of hormonal therapy. In contrast to the rapid onset of remissions observed after diabetes induction with alloxan,^64^ onset of remissions with diazoxide was delayed and began about 2 weeks after the start of treatment; the cause of this delay is unclear.

Treatment of the more aggressively growing MNU-induced mammary carcinoma of the rat with 300 mg/kg diazoxide given on 5 days/week induced a remission in 55% of the animals. This effect was completely abolished by additional treatment with 2 IU depot insulin/day.^200^ Thus, an insulin-mediated effect of diazoxide was proven.

Combined therapy with a low dose (75 mg/kg) of diazoxide and the alkylating agents melphalan or N-(2-chloroethyl)-N-nitrosocarbamoyl (CNC)-omega-lysine increased the therapeutic efficacy of both cytostatics up to twofold in the MNU-induced rat mammary carcinoma.^202^ However, after the end of treatment with diazoxide and alkylating agents, tumors in this group grew faster than in controls. In DMBA-induced rat mammary carcinomas, the combination of diazoxide and medroxyprogesterone acetate moderately increased the remission rate, but clearly shortened the remission duration.^203^ In contrast, adding 200 mg diazoxide per kg to 5 mg/kg tamoxifen synergistically prolonged the remission duration and decreased tumor weight, although the latter effect was lost at the higher dose of 50 mg/kg tamoxifen (Table 3).

In a clinical pilot study, diazoxide was used at a relatively low dose of 200–300 mg/day.^186^ For inclusion, the maximal tolerated fasting glucose level was 110 mg/dl, and 180 mg/dl after an oral glucose load with 75 g. Nine breast cancer patients were included, and the best response was seen in a 60-year-old woman, who had glucose levels of only 56–105–115 mg/dl after an oral glucose load. After progression of her cutaneous metastases during tamoxifen, she was supplemented with 200 mg diazoxide per day; fasting glucose levels rose to 90 mg/dl, and surprisingly tamoxifen-induced hyperhydrosis disappeared. Partial remission with this combination ended after 7 months, when liver metastases were detected sonographically. Two months later, both medicaments were withdrawn because of rapidly growing cutaneous metastases and pleural effusion. Another 2 months later, the patient exhibited a rebound response of 4 months duration with disappearance of pleural effusions, partial remission of the cutaneous metastases and stable size of the liver metastases. In two additional patients with prior disease progress, diazoxide treatment resulted in stable disease of 8 (combined with tamoxifen) and 4 months (monotherapy).^186^

*In vitro* studies have uncovered other mechanisms of action by which diazoxide suppressed proliferation of human acute leukemic T cells^204^ and growth of human lung cancer cells.^205^ However, the growth of human colon cancer cells^206^ and especially human glioma cells *in vitro*^207, 208^ and in nude mice^207^ was stimulated through diazoxide-mediated opening of K^+^-channels. The ATP-sensitive K^+^-channels were thereby found overexpressed in glioma cell lines U87 and U251, and human glioma tissue, and their opening by diazoxide stimulated cell cycle progression and proliferation through activation of the extracellular signal-regulated kinase pathway.^207^ In principle, diazoxide-associated hyperglycemia may also raise concerns about brain tumor stimulation given the well-established link between hyperglycemia and brain cancer progression.^17, 18, 23, 24^ However, chronic neonatal diazoxide therapy during postnatal days 2–12 did not induce any lesions or morphological changes of brain anatomy in mice,^209^ and to our knowledge no glioma or brain metastasis in humans has been reported after treatment with diazoxide.

## Conclusions

A large body of preclinical data has indicated that inhibition of the insulin/IGF-1 system has a therapeutic benefit for cancer-bearing animals. However, rodents and man differ in some aspects of their metabolic regulation in response to a certain diet or pharmaceutical intervention targeting the insulin/IGF-1 system. In animals, specifically DR in its various forms (CR/IF/STS, KD, protein restriction) has shown a potential for simultaneously targeting many of the pathways associated with insulin and IGF signaling, usually with no serious, or with even beneficial side effects such as a differential stress response between normal and tumor tissue.

We reviewed human data to obtain the following preliminary conclusions concerning insulin/IGF-1 modulation in humans: (i) DR could be considered as a supportive treatment during cancer therapy due to its probable antitumor effects, and due to its beneficial effects on human metabolism. Considering muscle mass maintenance and the putatively beneficial effects of ketosis, KDs and STS should be compared with chronic CR or protein restriction in clinical studies. (ii) Insulin-lowering drugs such as metformin and diazoxide provide another opportunity for improving cancer outcome in patients. Although their administration is probably easier accomplished than adherence to a DR regime, they do not mimic all the effects of DR. Similar to DR, their anti-neoplastic potential in humans is still insufficiently investigated. At least for metformin it can be expected that current clinical trials will catch upon this. Diazoxide was successfully studied in animals, exhibited first effects in a clinical pilot study and is worth to be further examined. The same may be true for insulin potentiation therapy. These approaches may be useful options in the ambition to exploit the full repertoire of insulin/IGF-1 modulation against cancer.

## Figures and Tables

**Table 1:** Metabolic effect of various dietary restriction regimes in C57BL/6 mice and insulin-resistant humans

*Parameter*	*FGF21*		*IGF-1*		*Insulin*		*Glucose*		*BHB*		*Body weight*	
*Species*	*Mouse*	*Human*	*Mouse*	*Human*	*Mouse*	*Human*	*Mouse*	*Human*	*Mouse*	*Human*	*Mouse*	*Human*
Starvation	↑	↑	↓	↓	↓	↓	↓	↓	↑	↑	↓	↓
Calorie restriction	↗a	↗a	↘a	↘a	↘ab	↘b	↘b	↘ab	↗b	↗b	↓	↓
Protein-deficient diet, isocaloric	↑	↑	↓	↓	↓	↑, —	↘b	↗b	—	—	↓	↑
Protein-deficient diet, hypocaloric	↑	↑	↓	↓	↓	↘b	↘b	↘b	—	—	↓	↓
KD, isocaloric	—	—	—	—	—	—, ↓	↑	—, ↓	↑	↑	↑	↓, —
KD, hypocaloric	↗a	↗ a	↘a	↘a	↓	↓	↓	↓	↑	↑	↓	↓

Abbreviations: BHB, β-hydroxybutyrate; FGF21, fibroblast growth factor 21; KD, ketogenic diet.

a, mainly dependent on the degree of protein restriction; b, mainly dependent on the degree of CHO restriction.

The protein-deficient diet is defined as a diet containing <10E% protein. The KD is assumed to contain >10E% protein.

**Table 2:** Clinical studies and case reports on various dietary restriction regimes during cancer treatment

*First author*	*Nebeling*^75^	*Safdie*^152^	*Zuccoli*^153^	*Chu-Shore*^154^	*Schmidt*^155^	*Fine*^76^	*Schroeder*^77^	*Champ*^156^	*Rieger*^157^	*Oshakbayev*^158^	*Schwartz*^159^
Publication year	1995	2009	2010	2010	2011	2012	2013	2014	2014	2014	2015
											
Number of patients	2	10	1	5	16	10	11	6	20	1	2
											
Age (years)	P1: 3. P2 (P2): 8.5	63 (44–78)	65	8.8 (2.6–47.3)	50.5 (30–65)	62 (52–73)	65 (50–86)	59.5 (34–62)	57 (30–72)	41	P1: 55. P2: 52
											
Tumor entity	P1: Stage IV anaplastic astrozytoma. P2: Stage III cerebellar astrozytoma	Various stage IIA–VI carcinomas of the breast (4), prostate (2), ovary (1), uterus (1), lung (1) and esophagus (1)	Glioblastoma multiforme	Tuberous sclerosis complex tumors, among others renal angiomyolipomas and subendymal giant cell tumors	Various stage IV carcinomas and metastases	Stage IV carcinomas of breast (*n*=2), colorectum (3), lung (2), ovary (1), esophagus (1) and fallopian tube (1)	Stage III and IV head and neck squamous cell carcinoma	Glioblastoma multiforme	Glioblastoma multiforme	Stage III–IV ovarian carcinoma (T3N2M1)	Glioblastoma multiforme
											
Diet type	KD	STS	CR-KD	KD	KD	KD	KD	KD (*n*=5), CR-KD (1)	KD	CR	CR-KD
											
Energy (kcal/d)	P2: 2 200	0	600	NA	NA	1236±161	NA	NA	NA	300–400	P1: ≈1900. P2: ≈1800
											
Diet duration	P1: 62 weeks. P2: 8 weeks	48–140 h before and 5–56 h after CT	8 months	40.2 (3–66) months	7 (0.4–12) weeks	27.5 (26–28) Tage	1–4 days	7.5 (3–12) months	6–8 weeks or until progress, respectively	6 months	
											
Concurrent therapy	P1: None. P2: Partly CT	CT	Partly RCT	Sirolismus (1)	None	None	None	RCT (*n*=4), adjuvant CT (2)	None	Physical activity of at least 10 000 steps per day	None
											
Body weight at diet initiation (kg)	P1: 11. P2: 24	NA	58	NA		71.5 (46.3–103.4)	NA	77.8 (64.4–130.2)	78.3±16.1	74	P1: 85. P2: 79
											
Body weight change (kg)	NA	P1 and P2: −3.2 kg. P4: −2.7 kg	−8 (−14%)	NA		−3.0±0.5 (−4.1±0.7%, *P*=0.08)	NA	−5.7±7.5 (−8.7±7.8%, *P*=0.05)	−1.86 (−2.2%, *P*<0,05)	−21 (−28%)	P1: −5(−6%). P2: −5 (−6%)
											
Early termination due to diet-related side effects	0	0	0	1 (subjectively perceived cognitive impairment)	4 (fatigue, familial problems)	0	0	0	3 (impairment of quality of life)	0	
											
Blood glucose (mg/dl) during diet (Change compared to baseline)	P1 and P2: 70–90	NA	63	NA	NA	NA (−3.2±3.7, NS)	NA	84±7.1 (↓, *P*=0.02)	92±9.1 (↓, NS)	49–95	P1: <80 (↓). P2: <100 (no change)
											
Ketone bodies (mmol/l) during diet	P1: 4.5–5; P2: 2–3	NA			NA					NA	P1+P2: 2–4
											
Validation of ketosis	Blood+urine	NA	Urine	Blood	Urine	Blood	NA	Urine+blood	Urine	NA	Blood
											
Diet-related side effects	None	P7: Grade I lightheadedness, drop in blood pressure	Mild hyperuricemia, mild hypoproteinemia	NA			None	Grade I constipation (*n*=2), grade I fatigue (4), grade II fatigue (1)		First 10–15 days: headache, nausea, ichorrhea from the genital tract, skin itching, muddy urine with occasional dysuria and fever up to 38.3 °C	P1: None. P2: Hypercholesterinemia, headache between week 6 and 8
											
Clinical result	P1: 21.77% less FDG uptake after 8 weeks, no change in tumor size on MRI scan, significant improvement in quality of life. Stability until 62 weeks follow-up. P2: 21.84% less FDG uptake after 8 weeks	General and substantial reduction in CT-related side effects compared with *ad libitum* diet. Low severity of CT-related side effects in four patients who fasted throughout all CT cycles. CT effects on tumor as expected.	After incomplete resection all MRI scans negative until end of diet; relapse 78 days after end of diet	Simultaneous occurrence of tumor growth, stagnation and new tumor formation in 3/3 children; stagnation in 2/2 adults; improvement of epileptic seizures in 3/5 patients	Only 5 of 16 patients able to adhere to diet until study end. These 5 had stable disease.	Significant correlation between stable disease or partial regression in FDG-PET and ketosis relative to baseline	Decrease of mean lactate concentration in tumors	Significantly lower blood glucose levels than in control group, even during glucocorticoid administration	No influence on course of disease	Stable disease	P1: Progress after 4 weeks. P2: Progress after 12 weeks.

Abbreviations: CR, calorie restriction; CT, chemotherapy; KD, ketogenic diet; NA, not available; NS, non-significant; P1, Patient 1; P2, Patient 2; RCT, radio-chemotherapy; STS, short-term starvation.

We tried to include all studies that gave an indication towards anticancer effects of the intervention.

**Table 3:** Combination therapy with tamoxifen and diazoxide of the DMBA-induced rat mammary carcinoma. (reproduced from ref. 203)

	*Tamoxifen 5 mg/kg*		*Tamoxifen 50 mg/kg*	
	*Monotherapy*	*+Diazoxide 200 mg/kg*	*Monotherapy*	*+Diazoxide 200 mg/kg*
Remission (%)	53	47	50	59
Tumor weight compared to controls (%)	75	49	31	39
Duration of remission (weeks)	7	12	8.5	12
